# Supplementary material for: Financing innovation and enterprises’ efficiency of technological innovation in the internet industry: Evidence from China
Source: PLoS One. 2020 Sep 15;15(9):e0239265. doi: 10.1371/journal.pone.0239265 (PMC7491745; doi:10.1371/journal.pone.0239265)
Supplement: Attachment — Submitted filename: Reviewers coms.docx [file pone.0239265.s002.docx]

Review Report for the manuscript titled: ***Financing innovation and enterprises’ efficiency of technological innovation in the internet industry:*** Evidence from China. I find the topic relevant and interesting for expanding current knowledge of the topic. However, several issues are a concern for me and they are presented below as thus;

Firstly, the authors need to state the source of the data for China’s digital economy being the second in size in the world after the United States. Also, authors need to develop a section in the work that brings out the gap they intend to fill. That particular aspect is lacking in the work.

Secondly, there should a clear cut section that deals with highlighting the hypothesis of the study. This is necessary in order to make the objectives of the study more eligible to readers.

Thirdly, it will be interesting to have a theoretical framework to back up study objectives, as this gives a clearer picture to the methodology developed in the other section of the work.

Furthermore, there should be clear reasons as to why the samples were divided into 58 financing innovations and 77 non-financing innovation enterprises. What is the motive for such division?

It is pertinent to show clearly the side of efficiency of technological innovation in the methodology section. Authors say they divided the samples into financing and non-financing enterprises, what measures show efficiency of technological innovation? That should be clearly stated in the methodology section.

The definition of efficiency of technological innovation on table 5 is not very clear. What does it mean and how was such variable measured? (Eq1-Eq6 doesn’t show enough meaning to the variable)

Lastly, the paper could benefit from a professional editing exercise.
