# Supplementary material for: Financing innovation and enterprises’ efficiency of technological innovation in the internet industry: Evidence from China
Source: PLoS One. 2020 Sep 15;15(9):e0239265. doi: 10.1371/journal.pone.0239265 (PMC7491745; doi:10.1371/journal.pone.0239265)
Supplement: Attachment — Submitted filename: Response to Reviewers.docx [file pone.0239265.s003.docx]

Dear Stefan and reviewers:

Thank you for your letter and the reviewers’ comments on our manuscript entitled “Financing innovation and enterprises’ efficiency of technological innovation in the internet industry: Evidence from China” (ID: PONE-D-20-12599). Those comments are very helpful for revising and improving our paper, as well as the important guiding significance to other research. We have studied the comments carefully and made corrections which we hope meet with approval. The main corrections are in the manuscript and the responds to the reviewers’ comments are as follows (the replies are highlighted in blue).

Replies to the reviewers’ comments:

1. The authors need to state the source of the data for China’s digital economy being the second in size in the world after the United States. Also, authors need to develop a section in the work that brings out the gap they intend to fill. That particular aspect is lacking in the work.

Response: We have added the source of data for China’s digital economy being the second in size in the world after the United States. Also, we have developed a section about the contributions of our work to state the gap we intend to fill.

2. There should a clear cut section that deals with highlighting the hypothesis of the study. This is necessary in order to make the objectives of the study more eligible to readers.

Response: We have added three hypotheses to construct a theoretical framework.

3. It will be interesting to have a theoretical framework to back up study objectives, as this gives a clearer picture to the methodology developed in the other section of the work.

Response: To answer this question, we have rewritten Chapter 2 “Literature review and hypothesis development”.

4. There should be clear reasons as to why the samples were divided into 58 financing innovations and 77 non-financing innovation enterprises. What is the motive for such division?

Response: The motive is detailed in the “Input and output variables” section.

5. It is pertinent to show clearly the side of efficiency of technological innovation in the methodology section. Authors say they divided the samples into financing and non-financing enterprises, what measures show efficiency of technological innovation? That should be clearly stated in the methodology section.

Response: The measures of technological innovation efficiency have been detailed in the methodology section.

6. The definition of efficiency of technological innovation on table 5 is not very clear. What does it mean and how was such variable measured? (Eq1-Eq6 doesn’t show enough meaning to the variable)

Response: The efficiency of technological innovation is calculated by Eq. (6). And the variables used in Eq. (6) have been introduced in Table 1.

7. The paper could benefit from a professional editing exercise.

Response: We found a professional editing organization for help. The certificate of language editing is uploaded with manuscripts.

Once again, thank you very much for your constructive comments and suggestions which would help us both in English and in depth to improve the quality of the paper.

Thank you and best regards.

Your sincerely,

Yueqin Lin

Corresponding author:

Name: Yueqin Lin

E-mail: adbhcew@163.com
